# Piezoelectric Hysteresis Modeling Under a Variable Frequency Based on a Committee Machine Approach

**DOI:** 10.3390/s25175371

**Published:** 2025-08-31

**Authors:** Francesco Aggogeri, Nicola Pellegrini

**Affiliations:** Department of Mechanical and Industrial Engineering, University of Brescia, Via Branze 38, 25123 Brescia, Italy; nicola.pellegrini@unibs.it

**Keywords:** hysteresis, Bouc–Wen, backlash, committee machine, genetic algorithm, particle swarm optimization

## Abstract

Piezoelectric actuators, widely used in micro-positioning and active control systems, show important hysteresis characteristics. In particular, the hysteresis contribution is a complex phenomenon that is difficult to model when the input amplitude and frequency are time-dependent. Existing dynamic physical models poorly describe the hysteresis influence of industrial mechatronic devices. This paper proposes a novel hybrid data-driven model based on the Bouc–Wen and backlash hysteresis formulations to appraise and compensate for the nonlinear effects. Firstly, the performance of the piezoelectric actuator was simulated and then tested in a complete representative domain, and then using the committee machine approach. Experimental campaigns were conducted to develop an algorithm that incorporated Bouc–Wen and backlash hysteresis parameters derived via genetic algorithm (GA) and particle swarm optimization (PSO) approaches for identification. These parameters were combined in a committee machine using a set of frequency clusters. The results obtained demonstrated an error reduction of 23.54% for the committee machine approach compared with the complete approach. The root mean square error (RMSE) was 0.42 µm, and the maximum absolute error (MAE) appraisal was close to 0.86 µm in the 150–250 Hz domain via the Bouc–Wen sub-model tuned with the genetic algorithm (GA).

## 1. Introduction

Piezoelectric materials have gained significant interest in recent years due to their distinctive ability to convert mechanical energy into electrical energy and vice versa. A wide range of applications, including precision actuators, sensors, energy-harvesting devices, and microelectromechanical systems (MEMS), have been investigated at different technological readiness levels [1,2]. The nonlinear and hysteretic behavior of piezoelectric materials presents significant challenges for accurate modeling and control, even as their use becomes increasingly widespread. The hysteresis effect, between input voltage and resulting mechanical displacement, is a predominant challenge in varying dynamic conditions such as frequency changes, which are typical in real-world applications [3,4].

Piezoelectric actuators offer significant force with minimal displacement, a unique characteristic that eliminates the need for moving parts. Piezoelectric actuators driven by voltage sources exhibit pronounced nonlinearities in comparison to charge-driven configurations. In most practical implementations, voltage amplifiers are the standard means of actuation. For that reason, their influence on the overall system response needs to be carefully considered in both modeling and control. Although the intrinsic electromechanical coupling of piezoelectric materials inherently defies the assumptions of linearity, linear models continue to be employed extensively in the current literature for controller synthesis and system validation purposes [5]. Traditional hysteresis modeling approaches—such as the Preisach model, Bouc–Wen model, and Jiles-Atherton framework [6]—have been employed extensively to describe the nonlinear behavior of piezoelectric actuators. The Preisach model has been generally accepted for its ability to represent rate-independent hysteresis with reasonable accuracy. Nevertheless, these models often rely on complex parameter identification procedures and may fail to generalize effectively across varying operational conditions, particularly under different frequency excitations. Physics-based hysteresis models are typically categorized into operator-based and differential-based approaches. Operator-based models, such as the Maxwell slip [7], Preisach [8], and Prandtl–Ishlinskii models [9,10,11,12], employ mechanical analogs or integral operators and are inherently rate-independent, neglecting frequency effects. Their structure complicates inverse control design. In contrast, differential-based models, including the Bouc–Wen and backlash-like formulations [13,14], utilize nonlinear differential equations, offering improved suitability for control implementation. Due to its balance of modeling reliability and computational tractability, the Bouc–Wen model was selected in this study to represent the hysteresis behavior of the target piezoelectric actuator system.

At the present time, machine learning (ML) techniques have emerged as powerful tools for modeling complex nonlinear systems, including hysteretic behavior in innovative materials. Neural networks, support vector machines, and recurrent architecture [15] have demonstrated success in capturing hysteresis loops with limited prior assumptions on physical behavior. However, most existing ML-based approaches focus on single model architectures trained under fixed conditions, which may lead to overfitting or poor generalization when the system operates under variable frequencies or multi-regime excitations.

To address these limitations, this study proposes a novel hybrid modeling strategy that combines physical and data-driven approaches, based on a committee machine (CM) framework, to predict the frequency-dependent hysteresis behavior of piezoelectric actuators. CM, or ensemble learning methods, combine multiple base learners to produce a composite predictive model with enhanced generalization and robustness. By aggregating the outputs of different learners, ensemble models reduce variance and overfitting, making them particularly effective for capturing complex and variable system dynamics in various applications [16,17,18]. Therefore, these methods could be advantageous in modeling nonlinear behaviors such as hysteresis, where traditional parametric models often fall short due to limited adaptability and scalability. For instance, research has demonstrated the efficacy of neural networks (NNs) and echo state networks (ESNs) for hysteresis modeling [19,20]. These approaches are often limited by their monolithic structure and lack of modularity. Moreover, conventional methods rarely incorporate dynamic features such as frequency-aware adaptation, which is essential for piezoelectric systems operating under variable excitation conditions. Recent advances in ensemble-based learning, including bagging, boosting, and stacking, have shown improved performance in modeling time-dependent nonlinear systems [21]. The adaptive ensemble techniques that adjust weights or learner contributions in response to system changes have proven effective in nonstationary environments. However, to the best of the authors’ knowledge, no prior work has systematically explored the use of ensemble-based machine learning frameworks in conjunction with frequency-aware adaptation mechanisms for modeling piezoelectric hysteresis. Our proposed approach addresses this gap by leveraging the diversity of ensemble members alongside a frequency-sensitive adaptation mechanism that enables dynamic reconfiguration of the learning system in response to excitation characteristics. In addition to improved accuracy and robustness, the proposed framework enhances interpretability through model modularity. Each learner within the ensemble can be analyzed independently, offering insights into frequency-specific dynamics and hysteresis behavior. This modular architecture also facilitates extensions to online learning and transfer learning paradigms, enabling deployment in real-time applications where continual adaptation is critical. Furthermore, ensemble learning is increasingly recognized as a powerful tool for physics-informed modeling of hysteresis-like systems, particularly when domain knowledge can be embedded into the base learners or fusion strategy [22]. These innovations position ensemble-based machine learning as a robust and extensible methodology for capturing the intricacies of piezoelectric hysteresis under dynamic operational conditions. The primary innovative contribution relies on the progress of traditional black-box ML models; the proposed CM procedure integrates multiple specialized sub-models trained across distinct frequency domains, enabling localized accuracy while preserving global consistency. The second contribution of the proposed model is that it dynamically adapts to new frequency regimes by leveraging a frequency-aware gating mechanism that selects or mixes the outputs of sub-models. This hybrid procedure allows the system to model both rate-independent and rate-dependent hysteretic behaviors without requiring explicit physical modeling. Finally, an extensive experimental validation was conducted using a piezoelectric actuator and a capacitive measurement system, demonstrating the benefits of adopting the proposed procedure in terms of obtaining accurate results and reducing training and deployment effort to assess hysteresis phenomena across varying frequencies.

## 2. Hybrid Procedure Based on Committee Machine Deployment

**Figure 1:**
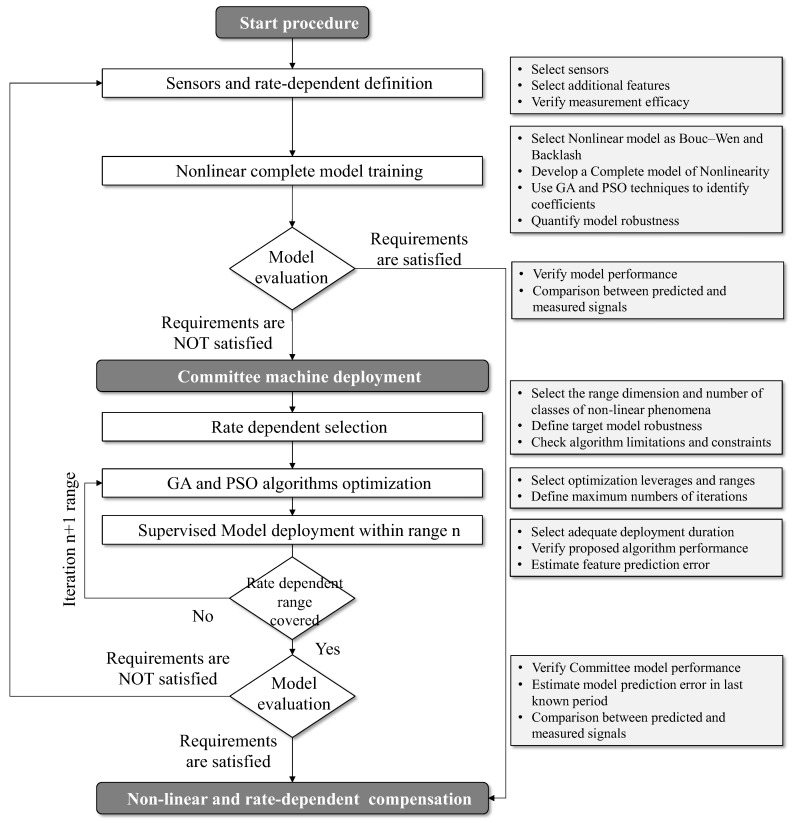
A hybrid modeling procedure that combines physical and data-driven approaches, based on a CM framework.

The proposed hybrid methodology for developing nonlinearities appraisal analysis is described in Figure 1. The procedure is designed to support iterative learning and progressive refinement of different physical and data-driven models, ensuring robust performance across a range of operational conditions characterized by hysteresis, unknown stage effects, and dynamic nonlinearities.

It starts by the definition of sensor architecture and the characterization of rate-dependent behavior. At this stage, the user identifies and configures the appropriate measurement instruments, ensuring that the selected sensors can capture relevant physical features under dynamic excitation. This includes the validation of measurement efficacy, ensuring that the subsequent data collected are reliable and suitable for model training. Additionally, this phase supports the extraction of temporal features that are instrumental in detecting rate-dependent effects. This point is particularly critical for a data-driven model approach. The analysis has to focus on data collection quality, the number and position of sensors, sensitivity, and sensor immunity to disturbances, cost, and complexity of installation. A model training phase follows, where a complete nonlinear model of the system is developed. This step involves selecting a modeling approach able to describe complex system dynamics. Typical candidates include physical models such as the Bouc–Wen formulation, known for its effectiveness in modeling hysteresis, or the backlash model, which captures mechanical slack and directional asymmetry. The model is trained using advanced optimization algorithms—genetic algorithms (GAs) and particle swarm optimization (PSO)—to identify the optimal parameters of the nonlinear system. The resulting model is evaluated through a performance validation phase. This evaluation assesses the model’s predictive ability by comparing the complete outputs against the measured data. If the model satisfies predefined accuracy criteria, it is considered suitable for deployment. Otherwise, the framework initiates an alternative strategy that involves deploying a CM. This ensemble learning method comprises a collection of local models that collaboratively provide predictions over subdivided portions of the input domain. The CM is particularly advantageous in dealing with systems whose behavior changes significantly across different ranges of input rates or amplitudes, enabling the compensation mechanism to adapt locally while maintaining global coherence.

**Figure 2:**
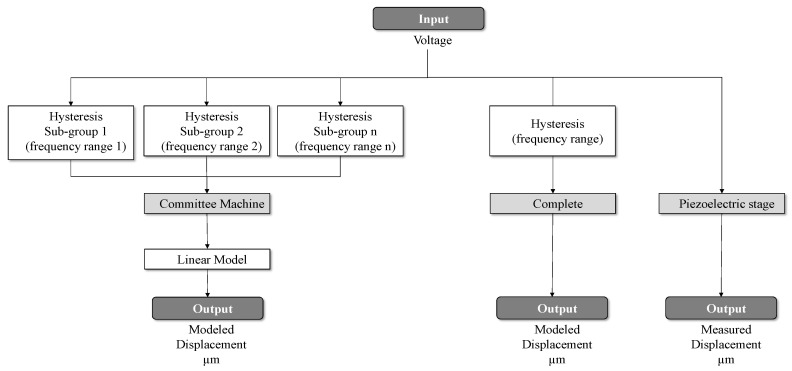
Graphical representation of the CM framework.

Then, the CM is initialized, and the framework states the rate-dependent selection phase, as schematically described in Figure 2. In this step, the operational domain is partitioned into multiple sub-regions based on the observed range of input rates. Each sub-region is assigned to a specific local model or learner within the ensemble, allowing for fine-grained model specialization. This decomposition also supports a targeted application of optimization routines, wherein each local model is further refined using the GA and PSO algorithms. These optimization procedures are configured with appropriate boundaries, iteration limits, and convergence thresholds to ensure computational efficiency while maintaining prediction accuracy.

After the optimization is complete, each sub-model undergoes a supervised deployment process. At this stage, the framework activates the model for a specified rate range and monitors its performance in real time. The supervised nature of this phase enables dynamic correction, model reweighting, and error estimation, thereby facilitating adaptive learning in a controlled environment. This is particularly important in scenarios where system dynamics evolve over time or under varying load conditions. After each deployment loop, the system evaluates whether the entire range of rate-dependent behavior has been adequately covered, considering that certain regions remain insufficiently modeled or exhibit unacceptable prediction errors. In that case, the process iteratively returns to the optimization phase for further refinement. This loop continues until all regions of the input domain are satisfactorily represented by the ensemble model. When full coverage has been achieved and the predictive accuracy of all models meets the required criteria, a final model evaluation is conducted. This final step verifies the performance by comparing its predictions to real measured outputs and estimating residual errors over the most recent operational period. If the ensemble model demonstrates sufficient generalization and stability, the final deployment phase is executed. The last stage of the framework is the implementation of nonlinear and rate-dependent compensation. Here, the hybrid procedure, via complete or committee approach, could be integrated into the system’s control architecture. The contribution represents a substantial improvement in control precision, reduction of phase lag, and minimization of steady-state and dynamic errors, which are essential for high-performance actuation in precision engineering applications. The proposed framework provides a rigorous, multi-stage methodology for modeling, validating, optimizing, and deploying adaptive compensation strategies for nonlinear and rate-dependent systems. Its architecture is modular, allowing for flexibility in model selection and scalability in deployment, making it suitable for a wide range of intelligent actuation and sensing scenarios in modern engineering systems.

## 3. Experimental Campaign and Results Comparison

The experimental campaign consisted of the adoption and characterization of a mechatronic device. The actuation was based on a piezoelectric stack element, PST 1000/25/40 VS35, as actuator, assembled in a metallic housing to the reference frame with a decoupling flexure axle to absorb the shear and torsional loads, while enabling the vertical motion of the end-effector. The active module had a maximum stroke capability of 40 µm, an axial stiffness of 450 N/µm, and a blocking force of 25 kN. These actuators require a corresponding power amplifier to reach both the high-frequency components and the high displacement (1000 V); hence, a high-power switching amplifier, RCV 1000/7, was employed for each line to avoid driving limitations and to limit the low-pass filter behavior of the power amplifier due to the high-capacitance actuator.

**Figure 3:**
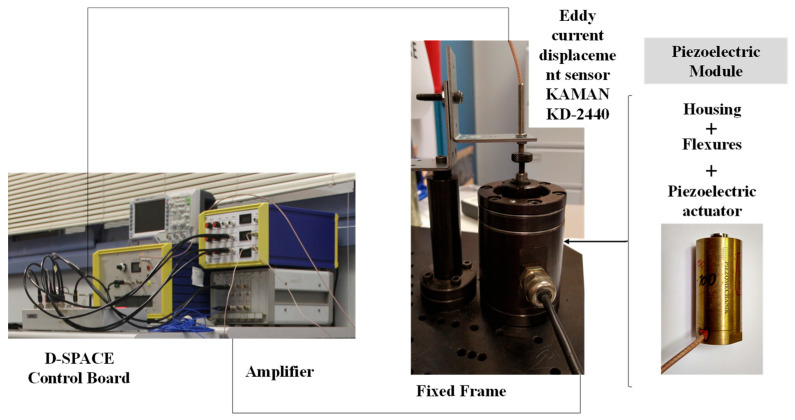
Experimental test bench: piezoelectric actuator module with measuring system and I/O connections.

Collocated on the end-effector, an eddy current displacement sensor, a KAMAN KD-2440 with a 5C probe, was positioned. The selection of this type of sensor was made due to its high precision, high bandwidth, compact electronics, and negligible hysteresis on ferrous targets. The dSPACE 1104 controller board was chosen to drive and receive signals from/to all the devices for the real-time measurement. The electronic loops were determined using a host PC running DSPACE and MATLAB/Simulink RTI environments. The driving analog voltages (±10 V) of the board were amplified to feed the corresponding input to the piezoelectric element, resulting in a consequent offset. During the closed-loop mode, the measured displacement with the eddy current sensor was acquired through the ADC port, and the output voltage was computed correspondingly for the synthesized controller loop. Furthermore, a graphical interface was produced in the dSPACE environment to assess the signals of interest and interact with the device. Figure 3 presents the schematic diagram representative of the hysteresis workbench and connections.

**Figure 4:**
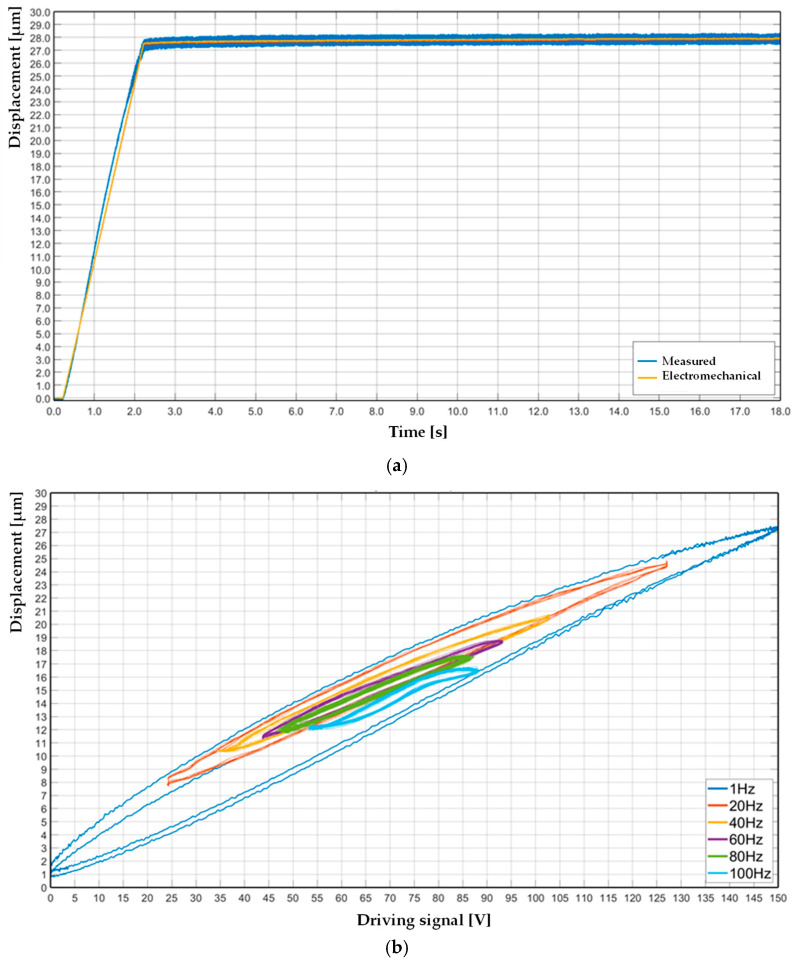
Nonlinear appraisal of piezoelectric actuator: creep effect, measured and modeled (**a**); measured hysteresis for multiple frequencies (**b**).

In the present work, the creep effect and the hysteresis contribution were experimentally measured for the complete piezoelectric actuation stage. The piezoelectric actuator drifted by creep phenomena of ~1.0 µm in 16.0 s, as in Figure 4a. The measured values from the experiments were compared with the output from the electromechanical model. The measured hysteresis at different frequencies is shown in Figure 4b within the range 1–100 Hz with an increment of 20 Hz. The actuator stage shows a significant influence of time-dependent characteristics in terms of displacement, min-max stroke, shape, and symmetrical behavior.

The modeling of hysteresis nonlinearities inherent in piezoelectric actuators using two distinct approaches, the Bouc–Wen model and the backlash model, using the MATLAB/Simulink 2024a RTI (real-time interface) refers to software frameworks that enable unified integration of Simulink models with real-time hardware and communication systems. These representations were employed to capture the complex, path-dependent behavior exhibited by piezoelectric materials under cyclic excitation, which significantly influences the control precision and system dynamics in high-resolution applications.

**Figure 5:**
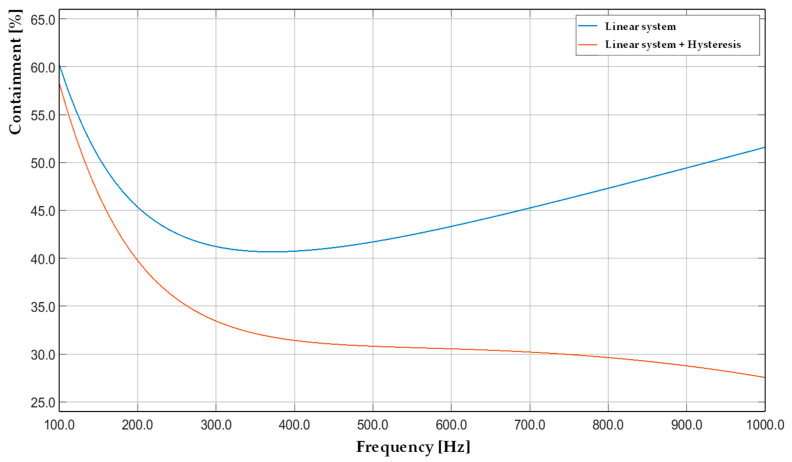
Closed-loop disturbance rejection simulation based on linear and physical-based containment percentage within the 100 Hz to 1.0 kHz range.

The Bouc–Wen model is widely adopted due to its ability to simulate continuous and smooth hysteretic loops by incorporating rate-dependent and memory effects through a set of nonlinear differential equations. The model’s parameters enable the tailoring of loop shape, stiffness degradation, and energy dissipation characteristics, which are crucial for accurately reflecting the dynamic response of the actuator across a broad frequency range. The backlash model offers a simplified, rate-independent characterization of hysteresis. Unlike the Bouc–Wen formulation, the backlash model emulates a discontinuous switching mechanism, analogous to mechanical backlash in transmission systems. It is less capable of capturing dynamic effects. However, it remains attractive for real-time implementations due to its computational simplicity and ease of parameterization. The comparison between the two models highlights the trade-off between accuracy and computational efficiency. While the Bouc–Wen model provides a more precise representation of the actuator’s nonlinear behavior, the backlash model may suffice for applications where model simplicity is prioritized. These models served as the basis for the subsequent optimization and validation stages. The simulated overall performance in terms of containment percentage is depicted in Figure 5. The blue curve represents the baseline: the linear system case. In this configuration, the minimum containment occurs in the 300–400 Hz range, reaching approximately 40% of the total displacement. In contrast, the maximum containment is observed at the boundaries of the frequency range, with 60% at 100 Hz and 51% at 1.0 kHz. These results were obtained without accounting for the nonlinear hysteresis contribution, which significantly impacted controller performance.

To address the limitation, the hysteresis impact was estimated using a basic low-pass filter model. In this case, the containment percentage decreased, especially at higher frequencies. The minimum containment was close to 28% at 1.0 kHz, while the maximum remained at 58% around 100 Hz. This modeling approach sustained the research motivation on hysteresis appraisal with a structured and scalable procedure. It is noted that this simplified model does not account for all nonlinear components associated with hysteresis. Consequently, when subjected to simulated validation tests using a more realistic model, the containment performance further degraded. In this final configuration, the minimum observed containment was lower than 30%, implying the impact of unmodeled nonlinearities on system performance.

### 3.1. Nonlinear Phenomena—Complete Model: Training and Deployment Results

To verify the proposed general model, the authors conducted the first experiment, evaluating the adoption of complete modeling with literature-recognized physical techniques, as implemented by optimization algorithms, in a wide range of real industrial contexts. Table 1 and Table 2 report the optimized parameters of the Bouc–Wen and backlash hysteresis models, respectively, obtained using two global optimization methods: genetic algorithm (GA) and particle swarm optimization (PSO). The optimization process was performed over a 1 h interval and repeated five times for robustness. The optimal parameter set was selected based on the minimum value of the integral of absolute error (IAE), as in Equation (1), computed over a [0.5–2.5 s] time window, ensuring consistent model performance over the target frequency range of 0–350 Hz.

(1) $$IAE=\int_{0.5}^{2.5}\left|y(t)-\hat{y}(t)\right|dt$$

In Table 1, the Bouc–Wen model parameters (α, β, γ, δ) and the associated filtering frequency are shown for each optimization method. GA produced a smoother nonlinearity (lower γ and δ) and a lower filter cutoff frequency than PSO, which may imply more effective compensation for signal distortion.

Table 2 summarizes the backlash model’s parameters (α, B_1_, c), reflecting its less complex structure. PSO notably drove B_1_ to zero, effectively simplifying the model form. These results established the basis for the complete model time-domain validation and comparison across frequency bands.

Table 3, Table 4, Table 5 and Table 6 present the performance metrics of both hysteresis models—Bouc–Wen and backlash—during the training phase. Integral absolute error (IAE) values (Table 3 and Table 5) are reported across four frequency intervals (0–50 Hz, 50–150 Hz, 150–250 Hz, 250–350 Hz) for both GA and PSO-optimized models. The Bouc–Wen model (Table 3) exhibited overall lower IAE values compared to the backlash model (Table 5), particularly in the higher frequency range, indicating its superior capacity to model rate-dependent dynamics. Maximum error values (Table 4 and Table 6), computed considering the sensor uncertainty margin of ±0.15 µm, further validated the accuracy of the models. While the GA-optimized Bouc–Wen model exhibited lower peak errors at high frequencies, PSO optimization showed minor errors in the mid-frequency range. Conversely, the backlash model showed less stability in error containment, with higher maxima observed mainly in the PSO case. These findings support the suitability of the Bouc–Wen model for applications involving broadband excitation.

**Figure 6:**
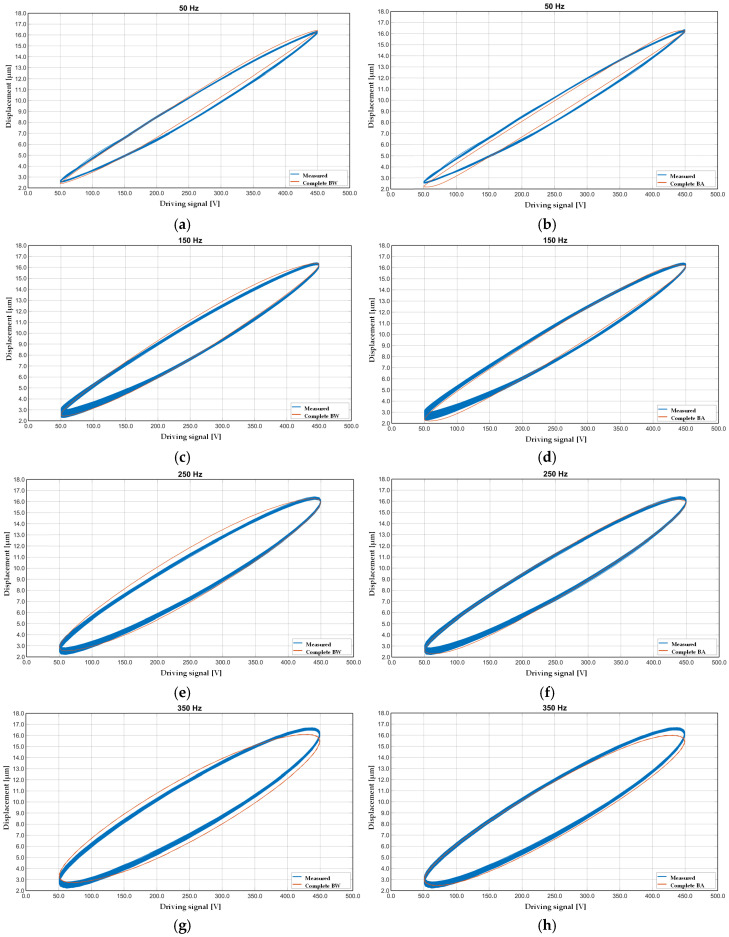
The hysteresis contribution at 50, 150, 250, and 350 Hz compared with complete Bouc–Wen model for (**a**,**c**,**e**,**g**), and the backlash model for (**b**,**d**,**f**,**h**), tuned with the GA algorithm.

Figure 6 illustrates the hysteresis behavior of two nonlinear models—the Bouc–Wen and the backlash models—across increasing excitation frequencies (50, 150, 250, and 350 Hz). Figure 6a,c,e,g show the Bouc–Wen response, while Figure 6b,d,f,h report the backlash model. Both models were optimized using a genetic algorithm (GA) due to the superior performance compared with the PSO from 3.5% to 26.9% in Bouc–Wen and backlash modeling, respectively.

**Figure 7:**
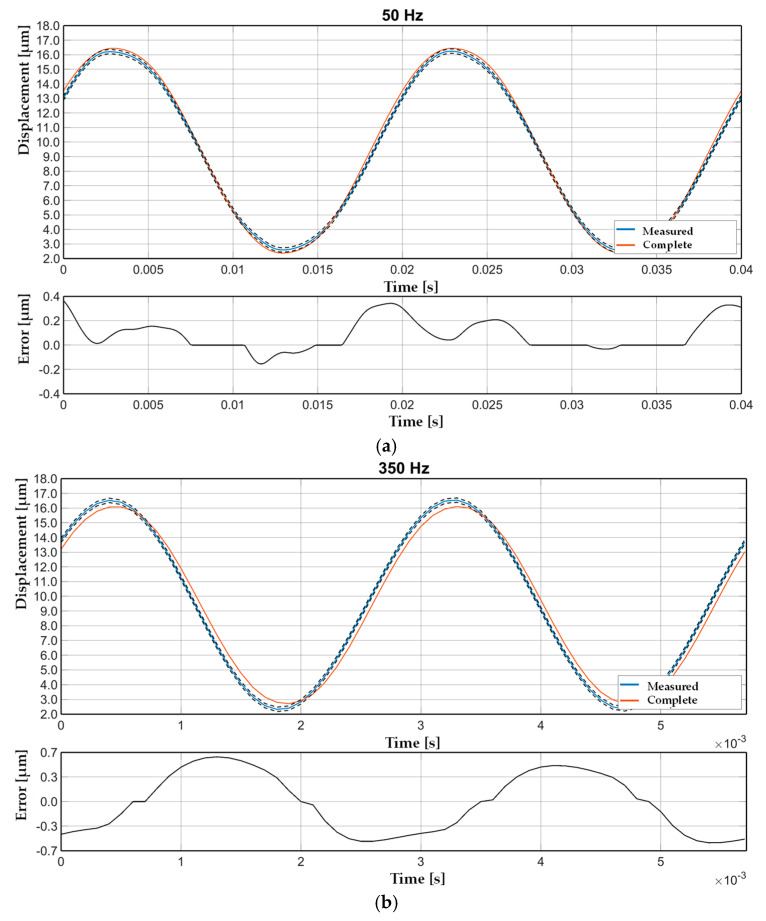
The Bouc–Wen complete model tuned with the GA algorithm compared with the measured piezoelectric actuator outcome.

At low frequencies, the hysteresis loops were similar, indicating minimal phase lag and rate-independent behavior. As the frequency increased, stated hysteresis developed, particularly in the Bouc–Wen model, which displayed smooth and continuous deformation of the loop, characteristic of its dynamic structure. The backlash model, while capable of reproducing basic nonlinearities, demonstrated a more sudden transition and loop flattening at high frequencies. Figure 7 presents a direct time-domain comparison between the output of the Bouc–Wen model (optimized via GA) and the measured displacement of the piezoelectric actuator under dynamic loading. The model output closely followed the measured data, capturing both the amplitude and phase characteristics of the actuator’s nonlinear response. The high-fidelity tracking of transient and steady-state behavior confirmed the model’s ability to generalize beyond the training dataset. Minor deviations were observed primarily during abrupt signal transitions, likely due to unmodeled dynamics or measurement noise. Nonetheless, the model remained compliant within the defined sensor uncertainty envelope (±0.15 µm as 95% confidence interval represented by dotted line) for the considered interval. The results visually validated the model’s performance in reproducing actuator behavior, reinforcing the quantitative results reported in Table 7 and Table 8.

Table 7, Table 8, Table 9 and Table 10 assess the generalization capabilities of the models by reporting the root mean square error (RMSE), in Equation (2), and maximum deviation in *µm* during the testing phase.

(2) $$RMSE=\sqrt{\frac{1}{n}\sum_{0}^{n}{\left(y(t)-\hat{y}(t)\right)}^{2}}$$

Table 7 presents the RMSE values of the Bouc–Wen model, where the GA-optimized version consistently outperformed PSO, particularly beyond 150 Hz. In Table 9, the backlash model presents higher RMSE values, particularly under PSO, confirming its relative limitations in capturing frequency-dependent hysteresis. Table 8 and Table 10 report the maximum error under the same frequency partitioning. Again, the Bouc–Wen model demonstrated a more stable error profile, especially when tuned via GA, with maximum deviations contained within ±1.22 µm at the highest frequency range. In contrast, the backlash model exhibited sharp increases in peak error under PSO, exceeding 1.15 µm at 250–350 Hz. These results validate the Bouc–Wen model’s greater robustness and fidelity in simulating piezoelectric actuator behavior under broadband dynamic excitation.

### 3.2. Noninear Phenomena—CM Model: Training and Deployment Results

Further experimental validation was carried out to validate the effectiveness of the proposed hybrid modeling of nonlinear phenomena, as presented in Section 2, in particular, the results of the parameter tuning and performance evaluation of the proposed CM-based modeling framework, applied to the characterization and compensation of nonlinear and rate-dependent hysteresis phenomena in the piezoelectric actuators stage. Two hysteresis formulations were selected: the Bouc–Wen and the backlash models, each optimized over four distinct frequency intervals (0–50 Hz, 50–150 Hz, 150–250 Hz, and 250–350 Hz) using two different heuristic techniques: genetic algorithm (GA) and particle swarm optimization (PSO).

Table 11 summarizes the identified parameters for the Bouc–Wen-based CM across the defined frequency sub-ranges. The reported parameters α, β, γ, δ, describe the internal dynamic behavior of the model. At the same time, the filter cutoff frequency quantifies the linear approximation bandwidth for model stabilization. Significant variations in parameter values across frequency intervals highlight the need for rate-aware identification, as model coefficients are significantly sensitive to frequency range. Table 12 presents the parameter configurations for the backlash-based CM. The α, B_1_, and c parameter values show the increasing stiffness and saturation characteristics with frequency, further supporting the necessity of frequency-dependent tuning. Differences between GA- and PSO-derived parameters explain the trade-offs between local and global optimization convergence.

To assess model accuracy during training, Table 13 reports the integral absolute error (IAE) for both Bouc–Wen and backlash configurations. The results indicate that, for the lower frequency band (0–50 Hz), both models achieve comparable performance. However, the Bouc–Wen model with PSO exhibited a reduced error profile across all bands, except in the 150–250 Hz interval, where the GA-backlash configuration proved more effective. Table 14 extends the evaluation by providing the maximum absolute error (MAE) during training. This metric shows the peak deviation between the model prediction and the target signal, offering insight into the worst-case modeling discrepancies. The backlash model tuned via GA exhibited higher peak errors in the low-frequency range but improved in the mid-to-high frequency bands, particularly when PSO was employed.

Validation in the testing phase is reported in Table 15 and Table 16, which contain the root mean square error (RMSE) and maximum error, respectively. The RMSE values in Table 15 confirm the findings from training: the Bouc–Wen model consistently outperformed the backlash model, particularly in lower and mid-frequency ranges. Conversely, Table 16 shows that the backlash model exhibited higher maximum deviations at high frequencies, when tuned via GA, facing model limitations in describing rate-dependent transitions in unknown operative conditions.

Based on the aggregate performance across training and testing phases, the optimal CM configuration is determined as follows:

- 0 Hz to 50 Hz: Bouc–Wen model tuned with PSO
- 50 Hz to 150 Hz: Bouc–Wen model tuned with GA
- 150 Hz to 250 Hz: backlash model tuned with GA
- 250 Hz to 350 Hz: Bouc–Wen model tuned with GA

**Figure 8:**
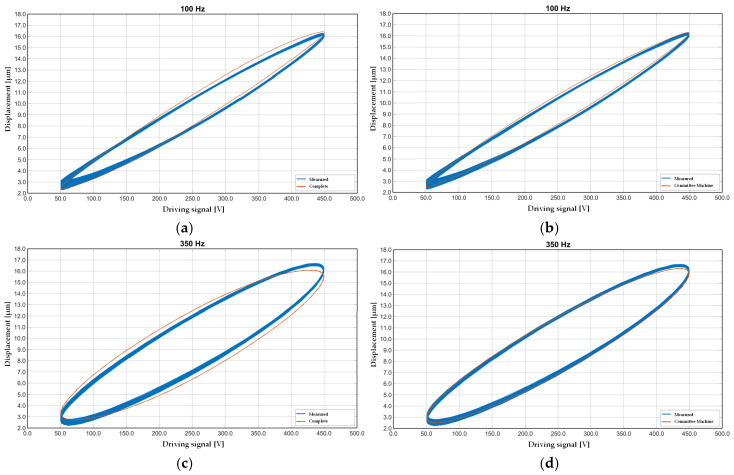
The hysteresis contribution at 100, 350 Hz compared with complete model (**a**,**c**) and CM (**b**,**d**).

This configuration represents the optimal balance between modeling accuracy and robustness across the considered frequency spectrum. Figure 8 provides a graphical comparison of the hysteresis effect modeled at two critical frequency points (100 Hz and 350 Hz), with the CM architecture. Figure 8a,c show the reference complete Bouc–Wen model tuned with the GA algorithm. At the same time, Figure 8b,d illustrate the results obtained by the CM under identical conditions.

In particular, Figure 8a,b highlight the model’s performance at 100 Hz, demonstrating the hybrid procedure’s ability to describe nonlinearity. The CM effectively reproduced the nonlinearity amplitude and phase lag, more accurately compared with the complete model output. Figure 8c,d focus on the higher-frequency case at 350 Hz, where rate-dependent effects enhance the dominance. Despite increased system complexity at this frequency, the CM continued to provide a satisfactory approximation. However, minor discrepancies in the loop shape and saturation points appeared, likely due to residual unmodeled dynamics or optimization bounds. Figure 8a–d confirm that the CM, when properly configured with frequency-optimized sub-models, can replicate the behavior of complex hysteretic systems with reduced computational effort and effective generalization capabilities.

## 4. Discussion

**Figure 9:**
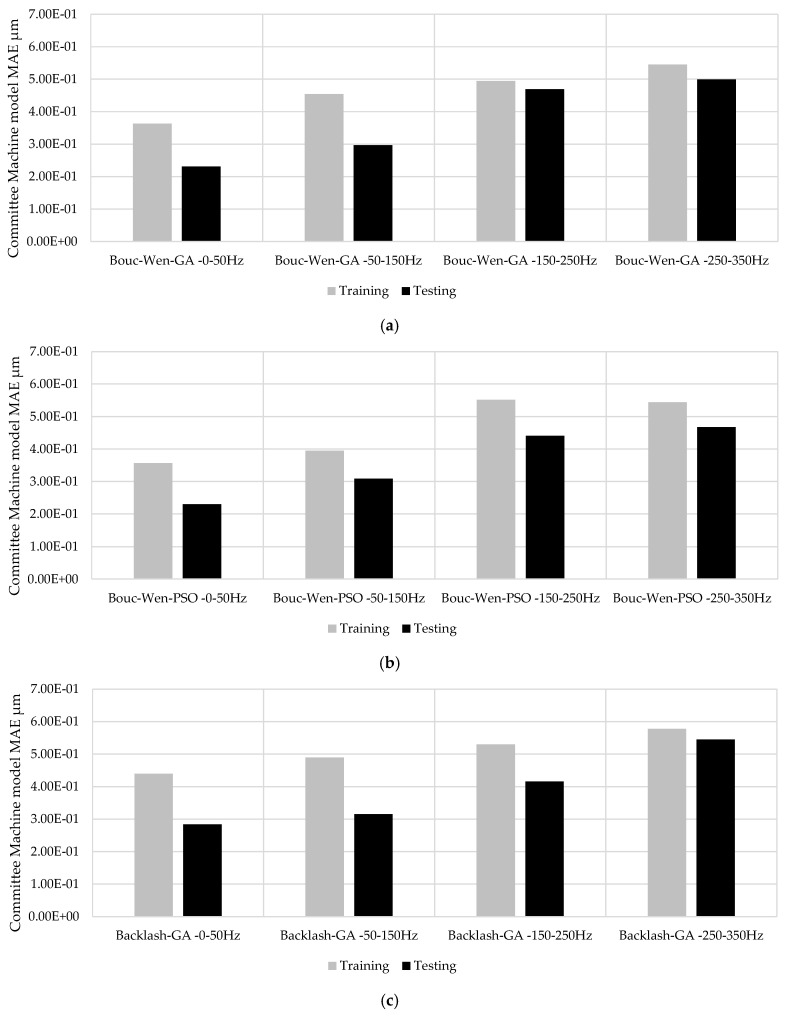

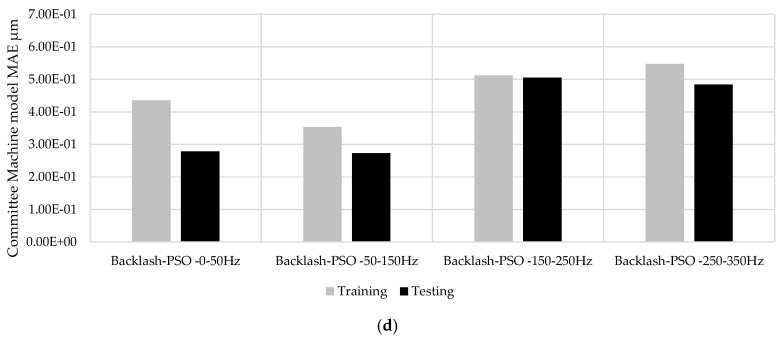
The testing–training ratio performance based on MAE as detailed in Table 14 and Table 16: Bouc–Wen GA, (**a**) Bouc–Wen PSO, (**b**) backlash GA, (**c**), backlash PSO, (**d**).

**Figure 10:**
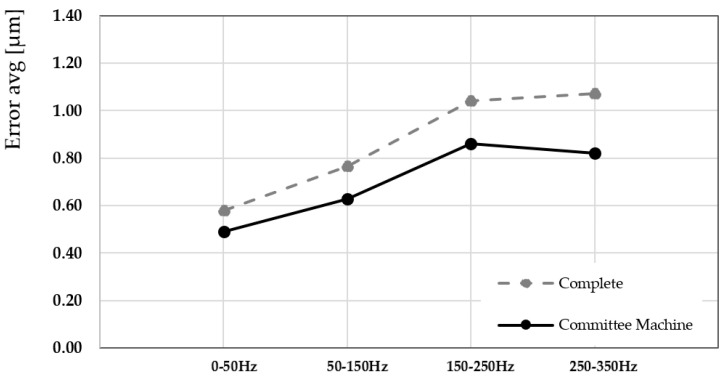
The average committee approach compared with the Bouc–Wen complete model tuned with the GA algorithm, supervised with the measured piezoelectric actuator outcome.

The proposed study highlights the following aspects:

- Piezo-actuated complex systems: the complexity of the mechatronic systems requires hybrid data-driven models to appraise the nonlinear effects. In high-precision applications, accurately modeling piezoelectric actuators is essential. Traditional approaches focus on the intrinsic nonlinearities and hysteresis of the piezoelectric material, which is inadequate for obtaining the actual behavior of a complete actuator system. Real-world industrial implementations integrate the piezoelectric element within mechanical structures, interfaces, preload assemblies, amplification mechanisms, and electronic drivers. These components introduce additional nonlinear dynamics, such as mechanical backlash, contact friction, structural compliance, and rate-dependent hysteresis effects. Neglecting system-level effects can lead to reduced prediction of actuator behavior, degraded control performance, and instability in feedback systems, especially at higher frequencies or under variable loads. The proposed procedure involves characterizing the overall mechatronic system or industrial equipment.
- Learning phase: the investigated frequency range of the learning phase influences the model selection. For this reason, a priori study is recommended to define the operative conditions for the piezo-actuator module stage usage. The proposed application aims to represent the common machining conditions of a 4-axis commercial machine tool, where piezo elements are increasingly adopted for workpiece positioning systems or active vibration absorber devices within the 300 Hz range.
- Data-driven model performance: the defined hysteresis conditions were modeled using the Bouc–Wen and backlash formulations, and parameters were identified through optimization methods, including genetic algorithm (GA) and particle swarm optimization (PSO). The optimization process was performed over a 1 h interval and repeated five times for robustness. The optimal parameter set was selected based on the minimum value of the integral of absolute error (IAE), computed over a [0.5–2.5 s] time window. The adopted method guarantees the trade-off between the convergence, the precision, and time-energy consumption. In industrial applications, the procedure needs to have minimal impact on setup time, thereby minimizing computational requests. The applied limitations in the learning phase could be improved by increasing data collection; nevertheless, continuous data collection and scalability to different mechatronics systems that operate concurrently may increase the model’s robustness. The obtained testing performance versus the training of the model is acceptable based on the percentage close to 64–65% in the <50 Hz frequency range and 86–92% at 250–350 Hz range as summarized in Figure 9a,d for the MAE index.
- Sensors: the use of an eddy-current sensor, with a precision of 0.3 µm and a 10 kHz bandwidth, affected the CM results. The output characteristics depend on both the supplied power and the properties of the target material. The element operated with a continuous supply voltage in the range of 12–24 V, which determined its maximum output signal. In the present setup, a stabilized Kert KAT4VD power supply was used to ensure consistent sensor performance.
- Results: this study shows a comparative evaluation of the proposed CM modeling strategy with the traditional single-model approaches. The Bouc–Wen and backlash complete models were selected for formulating the nonlinear hysteresis behavior of a piezoelectric actuator module. The analysis focused on accuracy, frequency adaptability, and error mitigation capabilities, and was validated against experimental measurements acquired from the real actuator system. Figure 10 illustrates a direct frequency-domain comparison between the output of the average CM and the Bouc–Wen complete model, both tuned via a genetic algorithm (GA), against the experimental response of the piezoelectric actuator. The CM integrated a frequency-partitioned modeling strategy, where distinct sub-models were trained and optimized for specific frequency intervals (0–50 Hz, 50–150 Hz, 150–250 Hz, and 250–350 Hz). The committee approach demonstrated a higher degree of alignment with the measured data across the entire excitation cycle. Particularly in regions characterized by rapid input variations and strong nonlinearities, such as transition phases and high-speed reversals, the committee model provided a more accurate description of the actuator’s dynamics than the Bouc–Wen model alone. The complete model exhibited either overshooting or phase lag under varying frequency conditions, highlighting the advantage of the modular design of the committee approach.

**Figure 11:**
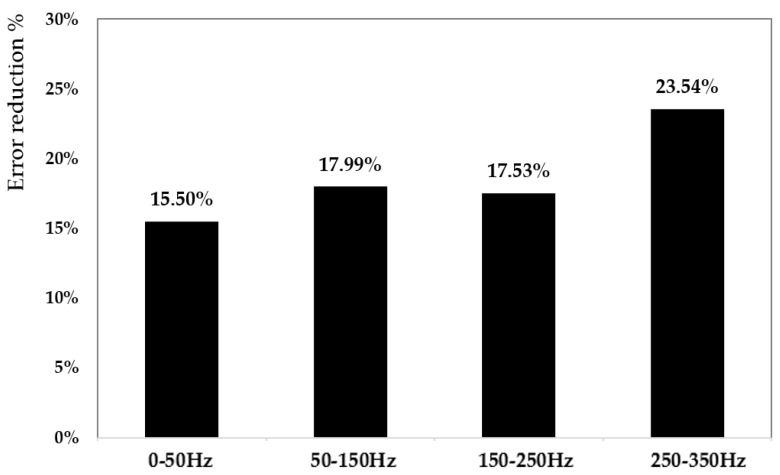
The error reduction percentage comparison between committee and complete Bouc–Wen models tuned with the GA algorithm.

**Figure 12:**
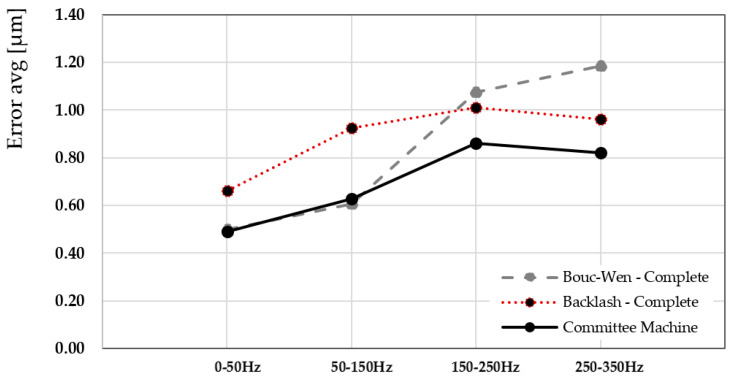
The average committee approach compared with the Bouc–Wen complete model tuned with the GA algorithm and the backlash complete model tuned with the GA algorithm, supervised with the measured piezoelectric actuator outcome.

Figure 11 presents a quantitative evaluation of the performance gain achieved by utilizing the CM. The figure shows the percentage reduction in modeling error compared to the Bouc–Wen complete model (also GA-tuned), evaluated using two error metrics: the root mean square error (RMSE) and the maximum absolute error (MAE), over the complete frequency range. The CM provided improved results at 250–350 Hz frequency bands, where single models typically suffer from limited generalization due to fixed parameter configurations. The error reduction of 23.54% highlights the CM’s ability to mitigate overfitting by assigning specialized models to frequency segments where their performance is maximized. This also demonstrates that hybrid architecture ensured better representation of both low-frequency (50–50 Hz) quasi-static behavior and high-frequency dynamic transients. Figure 12 extends the comparison by including the Bouc–Wen complete model and the backlash complete model, both optimized using the GA algorithm. The figure compares their respective time-domain responses against the average CM model and the measured actuator data. The inclusion of both traditional models provides a holistic view of their strengths and limitations. The Bouc–Wen model is better suited to describing smooth transitions due to its differential formulation. In contrast, the backlash model manages rapid saturation and dead-zone phenomena. The CM, by integrating and weighing these behaviors across sub-modules, offered a balanced and robust output. It proved a maximum error of 0.86 µm to the actual measured behavior during the actuation cycle, including peak amplitudes, steady-state values, and transient dynamics. This further validated the effectiveness of the CM in synthesizing the strengths of different nonlinear modeling paradigms. The experimental tests demonstrate superior accuracy, adaptability, and generalization performance of the CM approach for modeling piezoelectric hysteresis. By leveraging a multi-model ensemble strategy, it overcomes the limitations of conventional single-model structures and provides a highly reliable tool for predictive modeling in complex, nonlinear systems.

## 5. Conclusions

This paper proposes a hybrid procedure that combines a physical and data-driven model to describe the hysteresis, the unknown effects, and dynamic nonlinearities of a mechatronic piezo-actuated system. These piezoelectric materials are becoming a practical solution; nevertheless, their intrinsic nonlinearities and hysteresis behavior, in addition to the mechanical structures’ interaction, preload assemblies, and electronic drivers, may generate undesired effects. These components may introduce additional mechanical backlash, contact friction, structural compliance, and rate-dependent hysteresis contributions. At the system level, this can lead to reduced prediction of actuator behavior, degraded control performance, and instability in feedback systems, in particular at higher frequencies or under variable loads. This study focuses on a piezoelectric stack element, PST 1000/25/40 VS35, as actuator, assembled in a metallic housing to the reference frame with a decoupling flexure axle to absorb the shear and torsional loads, while enabling the vertical motion of the end-effector. The active mechatronic module has a maximum stroke capability of 40 µm, an axial stiffness of 450 N/µm, and a blocking force of 25 kN. The modeling of hysteresis nonlinearities inherent in piezoelectric actuators considered two distinct approaches: the Bouc–Wen model and the backlash model. Starting with the complete representation, the procedure identified the model parameters based on the settled test bench using two global optimization methods: genetic algorithm (GA) and particle swarm optimization (PSO). The optimization process was performed over a 1 h interval and repeated five times for robustness. The optimal parameter set was selected based on the minimum value of the integral of absolute error (IAE), computed over a [0.5–2.5 s] time window, ensuring consistent model performance over the target frequency range of 0–350 Hz. The results show a maximum error during the testing phase of 1.15 µm for the backlash model tuned via the PSO technique, which is not compliant. This error was reduced to 1.05 µm with the Bouc–Wen model from the actual measured behavior during the actuation cycle, including peak amplitudes, steady-state values, and transient dynamics. The adopted method aims to guarantee the trade-off between the convergence, the precision, and time-energy consumption. In industrial applications, the procedure needs to have minimal impact on setup time, thereby minimizing computational requests. In the light of these considerations, the hysteretic nonlinear error may be correctly modeled using a data-driven CM approach. Two hysteresis formulations were chosen: the Bouc–Wen and the backlash model, each optimized over four distinct frequency intervals (0–50 Hz, 50–150 Hz, 150–250 Hz, and 250–350 Hz) using two different optimization techniques: genetic algorithm (GA) and particle swarm optimization (PSO). Based on the aggregate performance across training and testing phases, the optimal CM configuration is determined as follows:

- 0 Hz to 50 Hz: Bouc–Wen model tuned with PSO
- 50 Hz to 150 Hz: Bouc–Wen model tuned with GA
- 150 Hz to 250 Hz: backlash model tuned with GA
- 250 Hz to 350 Hz: Bouc–Wen model tuned with GA

The proposed procedure offers the most promising properties to nonlinear modeling under varying frequency conditions. This study shows that the error reduction of 23.54% for the CM confirms the ability to mitigate overfitting by assigning specialized models to frequency segments where their performance is maximized. The residual maximum error of 0.86 µm in the 150–250 Hz domain is appraised via the Bouc–Wen sub-model tuned with GA.

Future developments may consider enhancing the explainability and interpretability of the hybrid procedure; specifically, extending it to online learning and deploying transfer learning settings will be explored to enable complete generalization and scalability in real-world domains.

## Figures and Tables

**Table 1:** Bouc–Wen complete model parameters.

Optimization	Frequency Interval	α	β	γ	δ	Filter Frequency
GA	0 Hz to 350 Hz	−2.580 × 10^−1^	1.458 × 10^−2^	−2.274 × 10^−3^	7.384 × 10^−3^	7.415 × 10^3^
PSO	0 Hz to 350 Hz	−4.394 × 10^−1^	8.660 × 10^−3^	−2.476 × 10^−2^	5.345 × 10^−2^	9.700 × 10^3^

**Table 2:** Backlash complete model parameters.

Optimization	Frequency Interval	α	B_1_	c
GA	0 Hz to 350 Hz	4.057 × 10^−2^	1.956 × 10^−1^	9.839 × 10^−1^
PSO	0 Hz to 350 Hz	5.937 × 10^−2^	0.000	9.784 × 10^−1^

**Table 3:** Integral absolute error of Bouc–Wen complete model—training phase.

Model	Optimization	0–50 Hz	50–150 Hz	150–250 Hz	250–350 Hz
Bouc–Wen	GA	4.19 × 10^−1^	4.57 × 10^−1^	6.22 × 10^−1^	7.57 × 10^−1^
Bouc–Wen	PSO	5.01 × 10^−1^	5.29 × 10^−1^	6.13 × 10^−1^	6.75 × 10^−1^

**Table 4:** Maximum error of Bouc–Wen complete model—training phase.

Model	Optimization	0–50 Hz	50–150 Hz	150–250 Hz	250–350 Hz
Bouc–Wen	GA	4.54 × 10^−1^	5.76 × 10^−1^	1.08	1.20
Bouc–Wen	PSO	5.31 × 10^−1^	6.25 × 10^−1^	1.03	1.12

**Table 5:** Integral absolute error of backlash complete model—training phase.

Model	Optimization	0–50 Hz	50–150 Hz	150–250 Hz	250–350 Hz
Backlash	GA	6.06 × 10^−1^	5.65 × 10^−1^	5.07 × 10^−1^	6.54 × 10^−1^
Backlash	PSO	6.96 × 10^−1^	7.89 × 10^−1^	8.00 × 10^−1^	6.62 × 10^−1^

**Table 6:** Maximum error of backlash complete model—training phase.

Model	Optimization	0–50 Hz	50–150 Hz	150–250 Hz	250–350 Hz
Backlash	GA	5.52 × 10^−1^	8.64 × 10^−1^	8.68 × 10^−1^	8.33 × 10^−1^
Backlash	PSO	7.63 × 10^−1^	9.81 × 10^−1^	1.14	1.04

**Table 7:** Bouc–Wen results complete RMSE—testing phase.

Model	Optimization	0–50 Hz	50–150 Hz	150–250 Hz	250–350 Hz
Bouc–Wen	GA	2.53 × 10^−1^	3.33 × 10^−1^	6.41 × 10^−1^	8.24 × 10^−1^
Bouc–Wen	PSO	2.94 × 10^−1^	3.45 × 10^−1^	6.05 × 10^−1^	7.55 × 10^−1^

**Table 8:** Bouc–Wen results complete max—testing phase.

Model	Optimization	0–50 Hz	50–150 Hz	150–250 Hz	250–350 Hz
Bouc–Wen	GA	4.61 × 10^−1^	5.82 × 10^−1^	1.10	1.22
Bouc–Wen	PSO	5.36 × 10^−1^	6.31 × 10^−1^	1.05	1.15

**Table 9:** Backlash results complete RMSE—testing phase.

Model	Optimization	0–50 Hz	50–150 Hz	150–250 Hz	250–350 Hz
Backlash	GA	3.28 × 10^−1^	3.12 × 10^−1^	4.52 × 10^−1^	5.78 × 10^−1^
Backlash	PSO	4.25 × 10^−1^	4.96 × 10^−1^	6.61 × 10^−1^	6.95 × 10^−1^

**Table 10:** Backlash results complete max—testing phase.

Model	Optimization	0–50 Hz	50–150 Hz	150–250 Hz	250–350 Hz
Backlash	GA	5.58 × 10^−1^	8.64 × 10^−1^	8.70 × 10^−1^	8.51 × 10^−1^
Backlash	PSO	7.67 × 10^−1^	9.86 × 10^−1^	1.15	1.07

**Table 11:** Bouc–Wen CM model parameters.

Optimization	Frequency Interval	α	β	γ	δ	Filter Frequency
GA	<50 Hz	−3.207 × 10^−1^	1.579 × 10^−2^	−5.564 × 10^−3^	1.850 × 10^−3^	9.616 × 10^3^
	50–150Hz	−2.385 × 10^−1^	5.059 × 10^−4^	−2.910 × 10^−2^	8.124 × 10^−2^	2.772 × 10^3^
	150–250 Hz	−2.483 × 10^−1^	8.985 × 10^−4^	−2.205 × 10^−2^	6.215 × 10^−3^	6.306 × 10^3^
	250–350 Hz	−4.497 × 10^−1^	1.084 × 10^−4^	−3.282 × 10^−2^	2.938 × 10^−2^	9.177 × 10^3^
PSO	<50 Hz	−2.564 × 10^−1^	1.247 × 10^−2^	−1.232 × 10^−2^	3.265 × 10^−7^	1.000 × 10^4^
	50–150 Hz	−3.045 × 10^−1^	9.368 × 10^−3^	−5.798 × 10^−3^	5.716 × 10^−2^	6.608 × 10^3^
	150–250 Hz	−2.991 × 10^−1^	4.867 × 10^−4^	−3.797 × 10^−2^	5.966 × 10^−2^	1.000 × 10^4^
	250–350 Hz	−1.889 × 10^−1^	5.328 × 10^−7^	−4.006 × 10^−2^	6.874 × 10^−2^	8.005 × 10^3^

**Table 12:** Backlash CM model parameters.

Optimization	Frequency Interval	α	B1	c
GA	<50 Hz	4.057 × 10^−2^	1.956 × 10^−1^	9.839 × 10^−1^
	50–150 Hz	4.693 × 10^−2^	4.069 × 10^−1^	9.635 × 10^−1^
	150–250 Hz	7.181 × 10^−2^	4.582 × 10^−1^	9.618 × 10^−1^
	250–350 Hz	2.924 × 10^−2^	7.076 × 10^−1^	9.997 × 10^−1^
PSO	<50 Hz	5.937 × 10^−2^	0.000	9.784 × 10^−1^
	50–150 Hz	1.824 × 10^−2^	6.638 × 10^−1^	9.733 × 10^−1^
	150–250 Hz	3.617 × 10^−2^	5.375 × 10^−1^	9.812 × 10^−1^
	250–350 Hz	2.533 × 10^−2^	7.751 × 10^−1^	9.852 × 10^−1^

**Table 13:** Integral absolute error of CM model—training phase.

Model	Optimization	0–50 Hz	50–150 Hz	150–250 Hz	250–350 Hz
Bouc–Wen	GA	3.63 × 10^−1^	4.54 × 10^−1^	4.95 × 10^−1^	5.45 × 10^−1^
	PSO	3.57 × 10^−1^	3.95 × 10^−1^	5.51 × 10^−1^	5.44 × 10^−1^
Backlash	GA	4.40 × 10^−1^	4.90 × 10^−1^	5.30 × 10^−1^	5.78 × 10^−1^
	PSO	4.36 × 10^−1^	3.54 × 10^−1^	5.12 × 10^−1^	5.47 × 10^−1^

**Table 14:** Maximum error CM model—training phase.

Model	Optimization	0–50 Hz	50–150 Hz	150–250 Hz	250–350 Hz
Bouc–Wen	GA	4.09 × 10^−1^	6.45 × 10^−1^	8.63 × 10^−1^	8.01 × 10^−1^
	PSO	4.01 × 10^−1^	5.33 × 10^−1^	8.25 × 10^−1^	7.56 × 10^−1^
Backlash	GA	5.51 × 10^−1^	8.29 × 10^−1^	8.55 × 10^−1^	8.61 × 10^−1^
	PSO	5.79 × 10^−1^	4.88 × 10^−1^	8.43 × 10^−1^	7.24 × 10^−1^

**Table 15:** CM results RMSE—testing phase.

Model	Optimization	0–50 Hz	50–150 Hz	150–250 Hz	250–350 Hz
Bouc–Wen	GA	2.31 × 10^−1^	2.97 × 10^−1^	4.69 × 10^−1^	4.99 × 10^−1^
	PSO	2.30 × 10^−1^	3.09 × 10^−1^	4.41 × 10^−1^	4.68 × 10^−1^
Backlash	GA	2.84 × 10^−1^	3.15 × 10^−1^	4.16 × 10^−1^	5.45 × 10^−1^
	PSO	2.78 × 10^−1^	2.73 × 10^−1^	5.05 × 10^−1^	4.84 × 10^−1^

**Table 16:** CM results max—testing phase.

Model	Optimization	0–50 Hz	50–150 Hz	150–250 Hz	250–350 Hz
Bouc–Wen	GA	4.10 × 10^−1^	6.48 × 10^−1^	8.94 × 10^−1^	7.39 × 10^−1^
	PSO	4.05 × 10^−1^	5.43 × 10^−1^	8.40 × 10^−1^	7.87 × 10^−1^
Backlash	GA	5.57 × 10^−1^	8.30 × 10^−1^	8.56 × 10^−1^	9.05 × 10^−1^
	PSO	5.90 × 10^−1^	4.91 × 10^−1^	8.49 × 10^−1^	7.50 × 10^−1^

## Data Availability

The dataset is available on request from the authors.
